# Genomics Insights into *Mycolicibacterium Hassiacum* Causing Infection in a Cat with Pyogranulomatous Dermatitis and Panniculitis

**DOI:** 10.3390/pathogens13090785

**Published:** 2024-09-11

**Authors:** Daniele Smedile, Manuela Iurescia, Virginia Carfora, Cristiano Cocumelli, Tiziana Palmerini, Elena Lavinia Diaconu, Ilaria Congiu, Valentina Donati, Fiorentino Stravino, Luigi Sorbara, Erica Romano, Andrea Caprioli, Antonio Battisti

**Affiliations:** 1 General Diagnostic Department, Istituto Zooprofilattico Sperimentale del Lazio e della Toscana “M. Aleandri”, 00178 Rome, Italy; daniele.smedile-esterno@izslt.it (D.S.); virginia.carfora@izslt.it (V.C.); cristiano.cocumelli@izslt.it (C.C.); tiziana.palmerini@izslt.it (T.P.); elena.diaconu@izslt.it (E.L.D.); ilaria.congiu-esterno@izslt.it (I.C.); valentina.donati@izslt.it (V.D.); fiorentino.stravino@izslt.it (F.S.); luigi.sorbara@izslt.it (L.S.); andrea.caprioli@izslt.it (A.C.); antonio.battisti@izslt.it (A.B.); 2 Veterinary Practitioner, 00165 Rome, Italy; ericaromano10@gmail.com

**Keywords:** *Mycolicibacterium hassiacum*, rapidly growing mycobacteria (RGM), mycobacterial panniculitis, cat, WGS

## Abstract

*Mycolicibacterium hassiacum* (homotypic synonym: *Mycobacterium hassiacum*) represents an ungrouped thermotolerant rapidly growing mycobacteria (RGM) species occasionally associated with infections and disease in humans. In this report, we describe a case of pyogranulomatous dermatitis and panniculitis due to *M. hassiacum* in an immunocompetent adult cat. To the best of our knowledge, this represents the first report of *M. hassiacum* infection in animals. We also report the results of the in-depth genome characterization of the isolate using a combined short- and long-read whole-genome sequencing (WGS) approach. We observed the lack of acquired-resistance genes and no evidence of mutations in housekeeping genes associated with resistance to rifampicin and isoniazid. We detected some virulence factors in our isolate, such as some associated with the interaction of mycobacteria with host cells, and the presence of multiple copies of heavy metal resistance genes (*ars*B, *ars*R, and *ars*L/*cad*L). In conclusion, *M. hassiacum* should be included among the RGM species associated with feline subcutaneous atypical mycobacteriosis (SAM). A reliable and fast RGM laboratory identification and characterization is important not only for an accurate etiological diagnosis but also for a correct approach to SAM treatment options.

## 1. Introduction

Rapidly growing mycobacteria (RGM) are a heterogeneous group of saprophytic Gram-positive, acid-fast bacilli that usually produce visible colonies on synthetic media within 7 days when cultured at temperatures ranging from 24 °C to 45 °C [1]. RGM are distributed ubiquitously in nature and can be commonly isolated from soil and waters, but can also behave as opportunistic pathogens in humans and animals. RGM are included in the wider group of nontuberculous mycobacteria (NTM), and in humans, they have been associated with skin, soft tissue, bone, and pulmonary infections, as well as disseminated diseases [2]. In household pets, and in particular cats, RGM have been associated with a clinical syndrome characterized by a chronic infection of the skin and subcutis, usually referred to as chronic panniculitis, which is also defined as “mycobacterial panniculitis” [3,4,5,6].

RGM are divided into six main phylogenetic groups: the *Mycolicibacterium chelonae–abscessus* group, the *M. mucogenicum* group, the *M. fortuitum* group, the *M. mageritense* group, the *M. wolinskyi* group, and the *M. smegmatis* group [7]. Another group includes the thermotolerant *M. thermoresistibile* and *M. flavescens*, capable of growing at temperatures up to 52 °C [8,9,10]. *M. hassiacum* represents an ungrouped thermotolerant RGM species and the most thermophilic member in the genus *Mycobacterium*, growing at temperatures up to 65 °C. *M. hassiacum* has only been rarely associated with cases of disease in humans [11,12], although there are also some reports of isolation from human samples of doubtful clinical significance [13,14,15]. To the best of the authors’ knowledge, the infection has never been described in animals. The objectives of the present study were to (i) describe a case of pyogranulomatous dermatitis and panniculitis caused by *M. hassiacum* in an immunocompetent adult cat; (ii) perform an in-depth genomics characterization of the *M. hassiacum* isolate by using a combined short- and long-read whole-genome sequencing (WGS) approach and bioinformatics, comparing it with other *M. hassiacum* genomes available in public repositories; and (iii) gain an insight into its pathogenic and zoonotic potential through the assessment of its main genomics traits (e.g., those associated with virulence and antimicrobial resistance).

## 2. Materials and Methods

### 2.1. Case History, Clinical Presentation, and Sample Collection

In October 2015, a five-year-old spayed female domestic longhair cat was presented with an anamnesis of approximately one-year history of cutaneous lesions characterized by multifocal nodules, ulcers, and fistulous tracts discharging a serous-hemorrhagic exudate. The patient was an indoor cat kept in Rome (central Italy) but, during summertime, usually brought to another house with free access to the garden and possible contact with other cats. The cat was negative for feline immunodeficiency virus (FIV) and feline leukemia virus (FeLV), regularly dewormed, and received core vaccines (against feline parvovirus, herpesvirus, and calicivirus) and treatments with ectoparasiticides. As reported by the owner, the cutaneous lesions appeared around one year earlier in the perianal region and progressively involved the groin, the inner and the outer side of the right thigh, and the back and the lumbar region. Reportedly, the cat had been empirically treated throughout the whole previous year with various therapeutic cycles of different antimicrobials, and one month before presentation to our attention, some cutaneous nodules were also surgically removed by the referring veterinarian. Despite the antimicrobial treatments and partial surgery, only limited improvement in the clinical picture was reported, and the lesions progressed.

At the time of presentation, the cat was still under treatment with marbofloxacin (2 mg/kg body weight per os, once a day). Physical examination revealed the presence of lymphoadenomegaly of the right popliteal lymph node, while dermatological examination revealed the presence of nodular/ulcerative dermatitis affecting the outer side of the right thigh. In particular, multiple plaques and cutaneous/subcutaneous nodules (from 0.5 to 1.5 cm diameter) with ulcers and draining fistulae were observed. The skin of the affected area (approximately 5 cm diameter) appeared thickened, alopecic, erythematous, hot, and painful but non-pruritic. In the inner side of the right thigh and the inguinal region multifocal alopecia, depigmentation and scarring were observed. A bioptic specimen was collected from one of the cutaneous lesions located on the outer side of the right thigh. The sample was aseptically divided into two portions: one was kept refrigerated and submitted for microbiological examination, while the other one was formalin-fixed for histology.

### 2.2. Routine Laboratory Analyses, Serology, and Histology

Trichoscopic examination for ectoparasites and Wood’s lamp skin test for dermatophytes were performed. Routine biochemical laboratory analyses, including hemogram, serum biochemical profile, and serum protein electrophoresis, were also performed after dermatological examination. *Toxoplasma gondii* antibodies (IgM and IgG) were tested by an immunofluorescence antibody test (IFAT). Histological sections of the biopsy were routinely processed and stained using hematoxylin and eosin (HE). Histochemical periodic acid–Schiff (PAS) and Ziehl–Neelsen (ZN) stains were also performed.

### 2.3. Bacterial Isolation

Bacterial cultures were performed by streaking the bioptic fresh portion onto duplicate Columbia agar supplemented with 5% sheep blood (bioMérieux, Marcy l’Etoile, France) plates, following incubation under aerobic conditions at 37 °C, and at 37 °C in the presence of 5–10% CO_2_. The specimen was also cultured onto two specific mycobacterium solid media, namely Lowenstein-Jensen and Coletsos–Ossein slanted culture tubes (Bio-Rad, Marne-la Coquette, France), following incubation under aerobic conditions at 37 °C. Columbia agar plates were evaluated daily for one week, while mycobacterium solid media were periodically evaluated for bacterial growth for up to three months, following the indications of the WOAH Manual of Diagnostic Tests and Vaccines for Terrestrial Animals 2022 [16].

### 2.4. 16S Molecular Analysis

#### 2.4.1. 16S rDNA Sequencing

DNA from suspect colonies referable to *Mycobacterium* spp. was extracted using the QIAamp DNA Mini Kit (Qiagen, Hilden, Germany) following the manufacturer’s protocol. DNA extracted from five selected colonies was tested by PCR, followed by the subsequent sequencing of 500 bp and 1500 bp target regions of the *16S* ribosomal DNA (rDNA) gene [17,18].

#### 2.4.2. 16S rDNA-Based Phylogenetic Analysis

All sequences were trimmed and aligned using ClustalW’s algorithm with default parameters and trimmed to obtain equal-length fragments as customary for phylogenetic analyses. A phylogenetic tree based on 16S rRNA encoding genes was generated using the Molecular Evolutionary Genetics Analysis software v11 (MEGA11) [19]. The maximum likelihood algorithm in combination with the Tamura 3-parameter model was used to assess the phylogenetic relationships between our *M. hassiacum* 16SrDNA sequence and other publicly available reference sequences belonging to different *Mycobacterium* species, including previously deposited *M. hassiacum* sequences (Table 1).

In particular, the following sequences were included in the phylogenetic analysis:

- A selection of 19 different RGM species closely related to *M. hassiacum* and previously described in a comprehensive phylogenetic study by Devulder et al. (2005) [20] on the *Mycobacterium* genus (including more than 90 *Mycobacterium* species);
- Two 16S rDNA publicly available sequences of two different *M. hassiacum* isolated in Germany from human clinical samples: one from urine (NR_026011.1 from strain 3849) [12] and one from sputum (MG386988 from strain JZ2014) [15];
- A 16S rDNA sequence obtained from the only publicly available *M. hassiacum* complete genome of approximately 5.3 Mb (NZ_LR026975 MHAS, strain DSM 44199) available at NCBI and published in 2019 [21], chosen as a reference for our study;
- A 16S rDNA sequence from *Nocardia abscessus* (AY544980.1, strain DSM 44432), selected as an outgroup for our phylogenetic analysis.

### 2.5. WGS and Bioinformatics Analysis

An in-depth characterization of our *M. hassiacum* isolate was performed by a combined short- and long-read WGS approach (Illumina-Oxford Nanopore Technologies, ONT) and subsequent bioinformatics analyses.

#### 2.5.1. Library Preparation and WGS

DNA extraction and library preparation were performed as previously reported [22]. Briefly, the library for short-read pair-end sequencing was prepared for the isolate using a Nextera XT DNA Library Preparation Kit (Illumina, Inc., San Diego, CA, USA) and sequenced on an Illumina platform (MiSeq sequencer, Illumina, Inc., San Diego, CA, USA). In parallel, a library from the same isolate was prepared with the ONT Rapid sequencing DNA V14-Barcoding Kit (SQK-RBK114.24) and sequenced using a nanopore-based MinION device (Oxford Nanopore Technologies, ONT, Oxford, United Kingdom) with an R10.4.1 flow cell, following the manufacturer’s specifications and the Super Accuracy basecalling model [23].

#### 2.5.2. “In Silico” Molecular Characterization

Illumina raw reads were analyzed using an internal pipeline. The quality of raw reads was evaluated using FastQC v0.11.5 and trimmed with TrimmomaticPE v0.22 [24] with the following parameters: Q30 as the minimum quality required for maintaining a base from the beginning and from the end of the read and a window size of 10 with Q20 as average quality. ONT reads and Illumina reads were assembled using the Unicycler (v0.5.0) [25] pipeline to resolve the genome of our isolate. The genome was hence annotated and manually curated using the online version of the BAKTA tool (https://bakta.computational.bio; accessed on 12 December 2023) [26]. The in-depth molecular characterization of our sequence was performed with the following bioinformatics tools and databases: (i) abricate 1.0.1 using the ResFinder database (*M. tuberculosis* scheme with 80% as the threshold for coverage and identity), the Comprehensive Antibiotic Resistance Database (CARD), and the AMRFinderPlus (version 3.11.17), and to detect the genetic basis of antimicrobial resistance (AMR), the online version of the Resistance Gene Identifier tool (RGI v6.0.3, https://card.mcmaster.ca/analyze/rgi, accessed on 12 December 2023) based on the CARD (v3.2.9) was also used with default parameters; (ii) the online tool VFanalyzer (http://www.mgc.ac.cn/VFs/, accessed on 5 March 2024), based on the virulence factor database (VFDB) for bacterial virulence factors detection, using the default parameters; and (iii) the online version of the CGE tool, MobileElementFinder v1.0.3 (https://cge.food.dtu.dk/services/MobileElementFinder/, accessed on 5 March 2024 [27] using default parameters, in order to identify Mobile Genomic Elements (MGEs) and their relation to AMR genes and virulence factors. PHAge Search Tool Enhanced Release (PHASTER) [28] was employed to identify and annotate possible prophage sequences within the bacterial genome.

A comparison between our genome and the NZ_LR026975 genome was conducted through CJ Bioscience’s Average Nucleotide Identity (ANI) [29] calculator and the Leibniz Institute DSMZ’s Genome to Genome Distance Calculator (GGDC) [30]. The first relies on the OrthoANIu algorithm, which uses USEARCH instead of BLAST [31]. Differently, GGDC computes identities considering the length of high-scoring segment pairs (HSPs) and estimates a DNA-DNA hybridization (DDH) of 97.20% [96.1–98%], based on its enhanced statistical models. The latter infers in silico whole-genome distances, which are well able to mimic wet-lab DDH(without mimicking its pitfalls) and yield higher correlations than the ANI software (https://anisoftware.wordpress.com/).

The alignment of our assembled genome with the NZ_LR026975 one was performed with the ProgressiveMauve algorithm [32] using default parameters and the Geneious software. Differences in the presence/absence of coding sequences (CDSs) were manually curated in both genomes, especially focusing on the presence of relevant AMR, toxigenic, virulence, and infective and phage-like genetic traits.

## 3. Results

### 3.1. Routine Laboratory Analyses, Serology, and Histology

The trichoscopic examination for ectoparasites and Wood’s lamp skin test for dermatophytes’ screening were both negative. The hemogram, serum biochemical profile, and serum protein electrophoresis were unremarkable. The cat also tested negative for *T*. *gondii* IgM and IgG antibodies.

**Figure 1:**
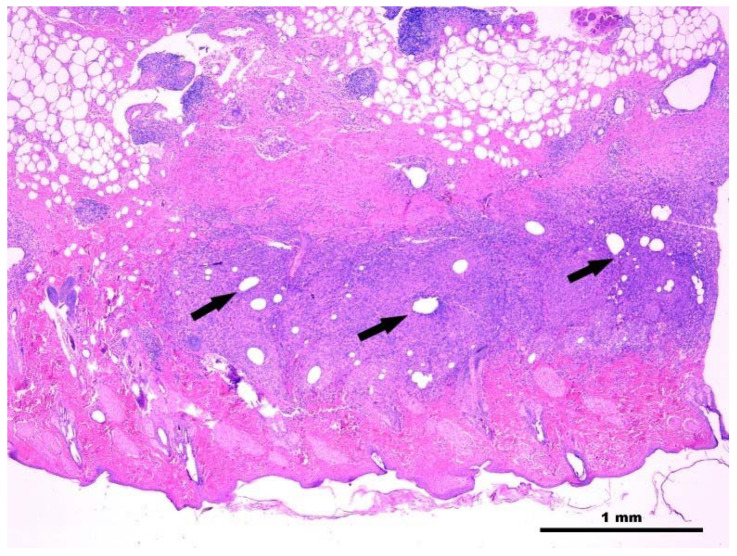
Histological section of a biopsy specimen collected from one of the cutaneous lesions. Multifocal to coalescing, deep pyogranulomatous dermatitis; granulomas (shown by arrows) are frequently centered around variably sized, optically empty vacuoles. HE stain, X40. Scale bar: 1 mm.

Following histopathology examination, a severe multinodular to coalescing pyogranulomatous deep dermatitis and panniculitis was observed, extending to the subcutis and involving the muscular layer. Granulomas were composed of a high number of segmented neutrophils, foamy macrophages, and epithelioid cells, with interspersed lymphocytes and plasma cells, as well as rare multinucleated giant cells. The center of the granulomas was frequently occupied by variably sized, optically empty vacuoles (Figure 1). The ZN stain did not reveal any acid-fast bacilli, and the PAS stain did not show any fungal elements.

### 3.2. Bacterial Isolation

After 6–7 days, pure cultures of rough, rubbery, and pale-yellow colonies were observed on the Columbia agar plate incubated at 37 °C in the presence of 5–10% CO_2_ and on both mycobacterium media, while on the Columbia agar plate incubated under aerobic conditions, colony growth was more dysgonic, although still referable to the same type. Colonies were all scotochromogenic since their yellow pigmentation increased with maturity and after exposition to light. The microscopic examination of Gram-stained culture smears revealed the presence of irregularly stained Gram-positive pleomorphic rods. These rods were also partially acid-fast by ZN staining.

### 3.3. 16S Molecular Analysis

#### 3.3.1. 16S rDNA Sequencing

The 500 bp target sequence showed a 471/471 bp homology with *M. hassiacum* and *M. buckleii* (GenBank, accession numbers NR026011.1 and AF000225.1, respectively), while the 1500 bp fragment sequencing led to the unambiguous identification (1189/1189 bp homology) of the sequence as *M. hassiacum* (GenBank, accession number NR026011.1).

#### 3.3.2. 16S rDNA-Based Phylogenetic Analysis

**Figure 2:**
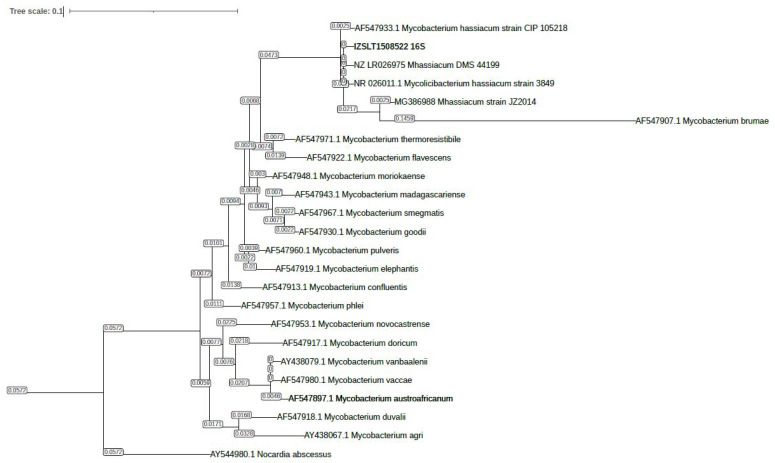
Phylogenetic tree based on the 23 selected 16S rDNA sequences belonging to different RGM species using a *Nocardia abscessus* sequence as an outgroup. Through the Best Phylogenetic Model Search Tool in MEGA11, the following parameters were selected: bootstrap method with 1000 replications, Tamura 3 parameter model + discrete GAMMA (+G = 0.1162), and maximum likelihood algorithm topology.

The overall structure of the phylogenetic tree constructed with the 23 selected 16S sequences was found to be comparable to the one described by Devulder et al. (2005) [20], also confirming the identification of our 16S sequence as belonging to *M. hassiacum* based on its localization in the phylogenetic tree (Figure 2). The alignment of sequences is shown in Table S1.

### 3.4. WGS and Bioinformatics Analysis

A closed chromosome of 4995820 bp and a 69.51% GC content were obtained from the hybrid assembly. The sequences were submitted to the European Nucleotide Archive (ENA) under study accession numbers ERR13402936 and ERR13402935 (raw reads’ ONT and Illumina, respectively).

Antimicrobial resistome prediction using RGI based on the CARD, uncovered various matches for AMR-related genetic sequences, with five of them showing higher values of coverage (range 99.06–100%) and identity (range 83.57–91.11%): (i) *mur*A (99.76% identity with 91.11% of coverage), a gene associated with intrinsic resistance to fosfomycin in *M. tuberculosis*; (ii) *rbp*A (100% identity with 85.59% of coverage) encoding a transcription factor associated with increased tolerance to rifampicin in mycobacteria; (iii) *arr-1* (99.30% identity with 83.57% of coverage) encoding a rifampin ADP-ribosyltransferase; and (iv) *kas*A (100%/86.54%) and (v) *rpoB* (99.06%/90.53%), the two housekeeping genes associated with resistance to rifampicin and isoniazid, respectively, when mutated.

A further comparison of the identified *rpo*B, via Clustal Omega, with the corresponding *M. tuberculosis* wild-type *rpo*B (A.N. NC_000962.3) revealed an identity value of 85.5%, showing no evidence of functional modifications in our gene. The analysis with the Resfinder database indicated that no acquired-resistance genes were present in our isolate.

Using VFDB [33], few virulence factors associated with the interaction of mycobacteria with host cells (Table S2) were detected in our samplethat were not detected by VFanalyzer in the *M. tuberculosis* H37Rv strain (NC_000962) used as a reference. In particular, our isolate harbored additional homologous sets of the mammalian cell entry operon such as *mce*5/7/8, which play a vital role in the entry of mycobacteria into the mammalian cell and their survival within phagocytes and epithelial cells. Recent findings showed the involvement of Mce proteins, among other cell-surface receptors, in the entry of these pathogens into the macrophages where they reside during infections [34]. In addition, genes encoding for glycopeptidolipids (GPLs) and two acid resistance ureases (*ure*B and *ure*C) were detected exclusively in our isolate, the former being responsible for the interaction between mycobacteria and human macrophages beyond their role in membrane composition, while the latter being involved in the production of large amounts of ammonia by hydrolysis of urea, as part of an acid resistance mechanism typical of Helicobacter species [35].

The MobileElementFinder tool identified the presence of two insertion sequences belonging to IS 1634 (coverage of 99.13 and identity of 85.72) and IS256 (coverage of 99.21% and identity of 87.94%) families and one putative transposable element (coverage of 95.47% and identity of 86.05%).

The PHASTER tool identified the presence of three prophage regions, with regions 1 and 2 (50.6Kb, position 967646..1018254 and 25.3Kb, position 4678616..4704004, respectively) remaining intact, while region three was incomplete (7.9Kb, position 4854168..4862150).

**Figure 3:**
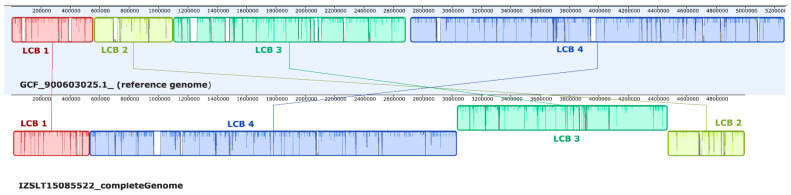
Comparison of our *M. hassiacum* genome with the reference one (NZ_LR026975) using the progressiveMauve algorithm.

Using the progressiveMAUVE algorithm (Figure 3), the comparison of our genome with the reference one (NZ_LR026975) highlighted a difference in a length of approximately 200 Kbp (our genome was 200 Kbp shorter than the reference).

Furthermore, using the OrthoANiu algorithm resulted in a 99.73% average identity. The results of BAKTA annotations of the two genomes showed differences in the general organization of the single chromosomes, which are highlighted in Table S3. The above-mentioned gene-by-gene analysis revealed that our genome differed from NZ_LR026975 for the presence or absence of specific transposable elements and phage-like genetic traits, such as virulence-associated protein-coding genes and genes coding for toxin–antitoxin systems such as RelE/ParE or heavy metal resistance genes (*ars*B, *ars*R, and *ars*L*/cad*L), which could be found in numerous copies throughout the genome.

## 4. Discussion

To the best of our knowledge, this represents the first report of *M*. *hassiacum* infection in animals. Since its first description in 1997 [13], *M. hassiacum* has been reported in a human patient with peritoneal dialysis peritonitis [11] and has been associated with pulmonary infection in an immunocompetent patient in Austria who had chronic obstructive pulmonary disease [12]. *M. hassiacum* has been also isolated from urine specimens of doubtful clinical significance [13,14] and has been isolated from a respiratory sample in a patient in Germany with the exacerbation of chronic obstructive pulmonary disease [15].

Our molecular investigation highlights that the poor availability of deposited sequences and the shortage of genera-specific databases referring to the various RGM species represent a clear challenge in the study of these sporadic pathogens. To achieve a precise identification and an in-depth characterization for diagnostic, research, and therapeutic purposes, there is a need to consult specific and periodically updated databases of relevant genetic determinants (e.g., AMR genes, virulence factors, etc.). For instance, although the *rpo*B (396 bp) gene phylogeny has been previously recommended to classify mycobacteria together with the *16S* rRNA gene-based one [20], this analysis could not be performed due to the lack of publicly available *M*. *hassiacum rpo*B sequences. An increasing number of deposited complete genomes, such as ours, that will be possibly used for comparison purposes and feeding curated ad hoc databases will be of help.

The RGM species previously associated with feline subcutaneous atypical mycobacteriosis (SAM) include several species [3,6,9,36,37,38,39,40,41,42,43,44,45], with the most representative being *M. fortuitum* [3,6,36,43], *M. smegmatis* [3,6,40,46] and *M. chelonae abscessus* [38,39,43,45]. As for the clinical presentation, the *M. hassiacum* infection reported in our case did not differ from other cases of reported feline SAM from other RGM [3,5,40,44]. The histological examination of biopsy specimens also showed typical features of feline SAM, such as the presence of a deep pyogranulomatous inflammation involving the subcutaneous adipose tissue and extending to the muscle layer [3,4,40]. It is remarkable that acid-fast bacilli were not observed in the ZN-stained tissues, as already reported [3,4,39]. In this regard, it has been previously hypothesized that the fixation process could affect the RGM capacity to take up or retain the stain [3,4,40]. In cats, the penetration of RGM into the subcutaneous tissues generally occurs through skin lesions following fight injuries or the penetration of foreign bodies [3,47]. In the present case, an initial traumatic event was not reported by owners, but it cannot be ruled out. As in the case herein described, SAM has been more frequently reported in spayed female cats. These cats are predisposed to obesity, and their fat deposits can facilitate the growth of RGM, which show a particular tropism for fatty tissues, such as the inguinal fat pad [3,5,43].

Regarding the treatment of mycobacterial panniculitis, only a few studies describe antimicrobial susceptibility testing (AST) of RGM isolates originating from animals [6,43,48,49,50]. The interpretation of these results is challenging because of a lack of published veterinary breakpoints. Previous studies reported prolonged antimicrobial therapies (on average ranging from several months to one year) often combined with an extensive surgical debridement/wound reconstruction when the removal of local skin lesions is feasible [3,43,51]. In any case, the use of antimicrobials following an accurate etiological diagnosis, and whenever feasible following a valid antimicrobial susceptibility testing, is essential to increase the probability of therapeutic success, limit the occurrence of ecological disturbances in the normal microbial flora, and reduce the risk of development and spread of resistances.

In our case, using a molecular approach, we observed the lack of acquired-resistance genes and no evidence of mutations in the housekeeping genes associated with resistance to rifampicin and isoniazid. In the future, this approach could also be used more to set up an appropriate therapy and to help overcome the challenges in the treatment of these infections.

At present, the zoonotic transmission of NTM, and in general of RGM, from household pets is considered possible but unlikely [52,53]. In any case, people living in contact with an infected household pet, especially in the case of immunocompromised owners [51], should always be informed about the potential zoonotic risk of mycobacterial infections. In this regard, the identification of some virulence factors in our isolate, such as those associated with the interaction of mycobacteria with host cells, and the presence of multiple copies of heavy metal resistance genes (*ars*B, *ars*R, and *ars*L*/cad*L) are features that may deserve attention whenever associated with further clinical cases in humans or animals.

## 5. Conclusions

In conclusion, *M. hassiacum* should be included among the RGM species associated with feline SAM. The incidence of SAM seems to be low, even if it could be underestimated due to vague or erroneous initial laboratory diagnoses. Possible misidentification with conventional cat-fight abscesses or with cutaneous/subcutaneous infections caused by other partly acid-fast bacteria (e.g., *Nocardia* spp.) should be considered [40]. A correct and fast RGM laboratory identification and characterization is important not only for an accurate etiological diagnosis but also for a correct approach to treatment options.

## Figures and Tables

**Table 1:** *Mycobacterium* species included in the phylogenetic analysis. All sequences used were partial sequences of the 16S ribosomal RNA encoding gene, except *Mycobacterium hassiacum* DMS 44199 (16S rRNA extracted from chromosome 1, complete sequence) and our sequence (IZSLT1508522; 16S rRNA extracted from complete genome).

	Accession Number	Organism
**1**	AY438067.1	*Mycobacterium agri*
**2**	AF547897.1	*Mycobacterium austroafricanum* strain CIP 105395
**3**	AF547907.1	*Mycobacterium brumae* strain CIP 103465
**4**	AF547913.1	*Mycobacterium confluentis* strain CIP 105510
**5**	AF547917.1	*Mycobacterium doricum* strain DSM 44339
**6**	AF547918.1	*Mycobacterium duvalii* strain CIP 104539
**7**	AF547919.1	*Mycobacterium elephantis* strain CIP 106831
**8**	AF547922.1	*Mycobacterium flavescens* strain CIP 104533
**9**	AF547930.1	*Mycobacterium goodii* strain CIP 106349
**10**	AF547943.1	*Mycobacterium madagascariense* strain CIP 104538
**11**	AF547948.1	*Mycobacterium moriokaense* strain CIP 105393
**12**	AF547953.1	*Mycobacterium novocastrense* strain CIP 105546
**13**	AF547957.1	*Mycobacterium phlei* strain CIP 105389
**14**	AF547960.1	*Mycobacterium pulveris* strain CIP 106804
**15**	AF547967.1	*Mycobacterium smegmatis* strain CIP 104444
**16**	AF547971.1	*Mycobacterium thermoresistibile* strain CIP 105390
**17**	AF547980.1	*Mycobacterium vaccae* strain CIP 105934
**18**	AY438079.1	*Mycobacterium vanbaalenii*
**19**	AF547933.1	*Mycobacterium hassiacum* strain CIP 105218
**20**	NR_026011.1	*Mycolicibacterium hassiacum* strain 3849
**21**	NZ_LR026975	*Mycobacterium hassiacum* DMS 44199
**22**	MG386988	*Mycobacterium hassiacum* strain JZ2014
**23**	**IZSLT1508522**	*Mycolicibacterium hassiacum*
**24**	AY544980.1	*Nocardia abscessus* strain DSM 44432

## Data Availability

Data supporting this study are included within the article and Supplementary Materials.

## Supplementary Materials

The following supporting information can be downloaded at: https://www.mdpi.com/article/10.3390/pathogens13090785/s1, Table S1: Alignment of 16S sequences. Table S2: VFDB *M. hassiacum* identified virulence factors. Table S3: Results of BAKTA annotation.
